# Latex-injected, non-decapitated, saturated salt method-embalmed cadaver technique development and application as a head and neck surgery training model

**DOI:** 10.1371/journal.pone.0262415

**Published:** 2022-01-20

**Authors:** Anuch Durongphan, Songsak Suksantilap, Nutthanun Panrong, Aimpat Aungsusiripong, Apipat Wiriya, Sasiprapa Pisittrakoonporn, Witchate Pichaisak, Benjaporn Pamornpol

**Affiliations:** 1 Department of Anatomy, Faculty of Medicine Siriraj Hospital, Mahidol University, Bangkok, Thailand; 2 Department of Otorhinolaryngology, Faculty of Medicine Siriraj Hospital, Mahidol University, Bangkok, Thailand; 3 Department of Orthopedic Surgery, Faculty of Medicine Siriraj Hospital, Mahidol University, Bangkok, Thailand; Flinders University, College of Medicine and Public Health, AUSTRALIA

## Abstract

Published cerebrovascular injection techniques have mostly used decapitated, fresh cadavers or heads embalmed with 10% formaldehyde. There have been no reports using vascular-injected cadavers for head and neck surgical training models or using vascular injections in saturated salt method-embalmed cadavers. Thus, we performed vascular labeling of five saturated salt method-embalmed cadavers without decapitation. Latex mixed with red ink was injected into the common carotid artery via a 3D-printed vascular adapter. The injection force was provided by a peristaltic pump. Thyroidectomy, submandibular gland excision, neck dissection, parotidectomy, and mandibulotomy were performed on both sides of each cadaver (n = 10). The consistency of the cadavers was softer than fresh ones. Subcutaneous tissues were well preserved, and muscles were moist and elastic. Five physicians graded the resemblance of the heads and necks of the latex-injected, saturated salt method-embalmed, non-decapitated of five cadavers compared to living humans using a Likert scale from 0 (no resemblance) to 5 (maximum resemblance). Fifty-two percent of the head and neck region resemblance scale ratings were four or five. Although the cadavers were practical for head and neck surgical simulations, the brain parenchyma was only partially preserved and unsuitable for use. The most distal arterial branches reached by the injected latex were measured. The external caliber of the smallest vessels reached were lacrimal arteries (mean caliber ± SD, 0.04 ± 0.04 mm; 95% CI [0, 0.09]). There were no significant differences in the mean caliber of the smallest vessels reached between the left- and right-sided arterial branches (all p < 0.05).

## Introduction

Vascular injection models enhance surgical training experiences for neurosurgery, microsurgery, general surgery, trauma surgery, and maxillofacial surgery [1–5]. At a microsurgical flap reconstruction training course, participants reported using a vascular injection model significantly improved their learning experience and their confidence rated by a pre-and post-course self-reported score (p < 0.005) [2].

Cadaver types used as injection models have been fresh, formaldehyde-fixed, Thiel’s method-embalmed cadavers. Cerebrovascular injection models have been mostly decapitated fresh cadavers or heads embalmed with 10% formaldehyde [1, 6–8]. However, this approach does not preserve the anatomical relationships of the neck because the suggested cut levels were at the fourth or the fifth cervical vertebra, while the cricoid cartilage’s inferior border is usually at the level of the seventh cervical vertebra [7, 9]. Thiel’s method-embalmed cadavers have been used in oral vascular studies [10–12]. Conventional formaldehyde-embalmed cadavers have been the least used in cranial and extracranial vascular studies [5, 13, 14]. There have been no reports using injected cadavers as head and neck surgical training models except for airway surgery simulations [15, 16]. Saturated salt embalming is a preservation method that uses a low formaldehyde concentration [17]. This cadaver type could serve as a training model for ultrasonography, central venous catheterization, thoracic surgery, and abdominal surgery [18]. There have been no reported studies of vascular injections in a saturated-salt solution cadaver.

The common vascular labeling materials are latex, silicone, and gelatin. We searched Medline, Google Scholar, and Google from 1946 up to January 2021 using the keywords “vascular injection technique,” “cerebral vascular injection,” “facial vascular injection,” and “neck vascular injection” and performed full articles review. We found 18 studies that used latex, nine studies that used silicone, and three studies that used gelatins. The injection volumes ranged from 100 to 180 mL per head [5, 7, 13, 19, 20]. The injection vehicles were Foley catheter, soft plastic tube, hypodermic syringe, plastic suction tubes, and nasogastric tube [1, 5, 7, 21]. The injection forces were manual with or without pressure monitoring and gavage [7, 11, 14, 21]. After cadavers were injected, they were typically kept for 24–72 hours before dissection [5, 19, 20]. Due to the heterogeneity of these published methods, standardization of the cadaver injection method and identification of the proper type of cadaver for high-fidelity surgical simulation are warranted. Thus, we aimed to study a low-cost, locally available, easily handled injection material to develop an injection technique for the cadaver with an intact head and body. Furthermore, we investigated the possibility of using latex-injected, saturated salt method-embalmed cadavers as head and neck surgical simulation models.

## Materials and methods

The institutional review board of Siriraj Hospital granted a certificate of approval (COA no. Si108/2020) for this research, which is fully compliant with the Declaration of Helsinki. Six legally donated cadavers were recruited. The cadavers were embalmed with a saturated salt solution by injecting the fluid using a manual pressure pump through the femoral artery. The solution contained 20% formaldehyde, phenol, glycerin, isopropyl alcohol, water, and pure sodium chloride dissolved in a mixture until saturated [18]. After that, the cadavers were stored in a separate plastic bag at room temperature for six months before the color injection process [17].

**Fig 1:**
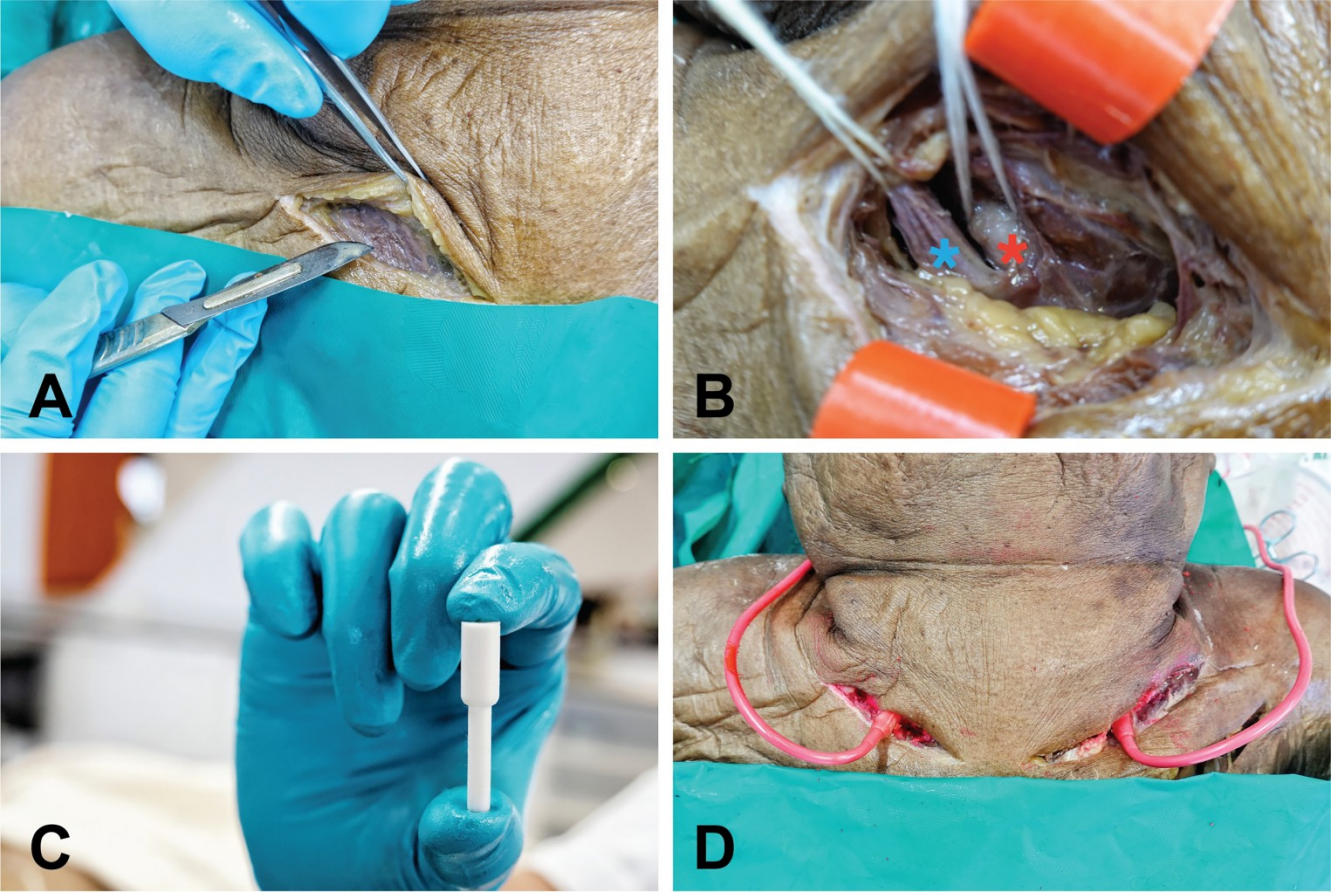
Injection step (A) Skin incision site at the upper border of the clavicle between the two heads of the sternocleidomastoid muscle. (B) Identification of the internal jugular vein (blue asterisk) and the common carotid artery (red asterisk). (C) The 3D-printed vascular adapter. (D) The injected mixture passing from one side to the other.

A three-dimensional vascular adapter (Fig 1) connected the common carotid artery (CCA) lumen with an extension tube. We designed an adapter with a computer-aided design program (Fusion 360, Autodesk, California, USA) and printed it via a fused deposition modeling printer (Original Prusa i3 MK3S, Prusa Research, Prague, Czech Republic). The printed material was polylactic acid plastic (Siamreprap, Bangkok, Thailand). The adapter vascular end’s external diameter ranged from 4 to 8 mm, similar to the known CCA luminal sizes [22].

The injection processes included injection site preparation, irrigation, injection, and curing. There was no decapitation step. A skin incision was made at the clavicle’s upper border between the two heads of the sternocleidomastoid muscle, similar to the landmark for internal jugular vein central line insertion [23]. The CCA was medial to the internal jugular vein. This process was done with minimal disturbance of the surrounding tissues to prevent injection material leakage. The right CCA was identified and distally ligated. Then a beveled incision was performed on the CCA anterior wall, and the posterior wall was left intact. The CCA luminal size was estimated, and then the appropriate size of the 3D-printed adapter was selected and inserted through the incision. The 3D vascular adapter needed to be fitted to the lumen to prevent any backflow of the injection material. An extension tube was connected to the other side, and the adapter was secured to the vessel by ligation. These processes were repeated on the left side. The head and neck vessels were irrigated with 300 mL of tap water. The injection materials were locally available natural latex (Rungart, Bangkok, Thailand) mixed with red ink (Rotring, Hamburg, Germany). The fluid was delivered on one side by a peristaltic pump (Kamoer 24V Automatic Water Peristaltic Pump, Kamoer Fluid Tech, Shanghai, China). The other side was left open to observe the flow. The designated volume of latex mixture to be injected was 150 mL. If there was no material flow from the observed side, another 30 mL was added. The injected volumes are listed in Table 1. When the injection process was finished, the cadavers were left for 48 hours for the latex to solidify.

**Table 1:** Demographic data and injection volumes.

Cadaver identification code	Gender	Age	Cause of death	Volume (mL)
A	Female	74	Aging	150
B	Male	69	Myocardial infarction	180
C	Female	80	Acute myocardial infarction	180
D	Female	63	Heart failure	180
E	Male	86	Pulmonary infection	150
F	Male	63	Hypertension	N/A

**Fig 2:**
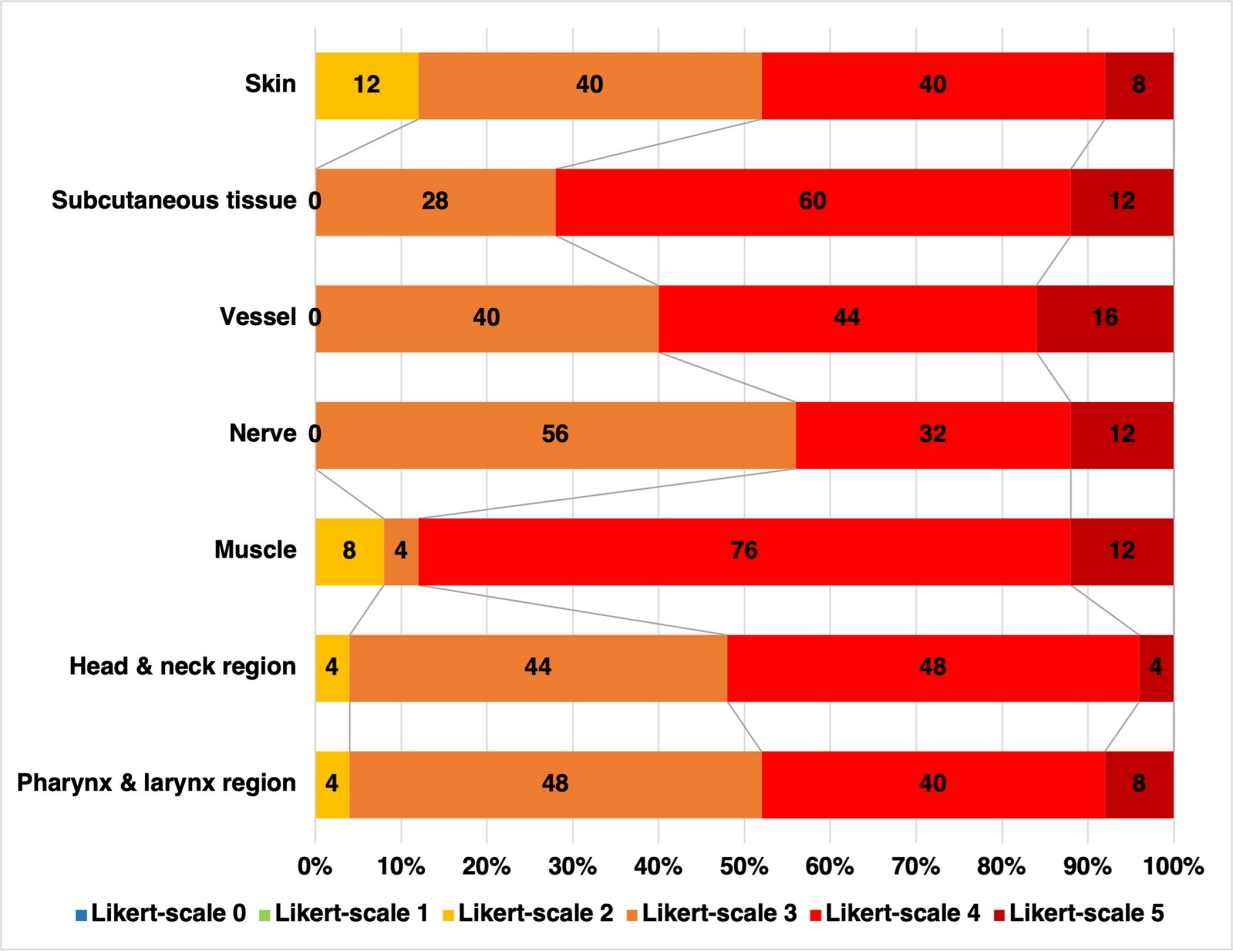
Likert scale ratings of the anatomical resemblance of 10 hemifaces and heminecks of five latex-injected, saturated salt method-embalmed cadavers compared to living humans graded by five surgeons. The Likert scale had six categories ranging from 0 (no resemblance) to 5 (maximum resemblance).

Head and neck surgical simulations were performed to evaluate the possibility of using the latex-injected, saturated salt method-embalmed cadavers as surgical training models. Thyroidectomy, submandibular gland excision, neck dissection, parotidectomy, and mandibulotomy were performed. The research team traced the head and neck vessels to verify the degree of filling of the injected latex mixture to document it using a Likert scale from zero to five. The definitions of the degrees of filling are in the S1 File, S1A Table. The most distal branches reached by the latex were recorded. Their external calibers were measured using a calibrated electronic Vernier caliper with a resolution 0.01 mm and an accuracy of ± 0.02 mm for structures < 100 mm). The mean of three measurements taken at each site was recorded (S1 File, S1B Table). The mean external caliber of each artery was calculated by grouping as left-side, right-side, and both sides (Table 2). The researchers were divided into an injection analysis team and a dissection measurement team to avoid bias. The dissection team was five physicians who had experience as surgeons. Each dissector graded the resemblance of the five latex-injected, saturated salt method-embalmed cadavers compared to living humans by using a Likert scale from 0 (no resemblance) to 5 (maximum resemblance). Fig 2 shows the frequencies and percentages of scale rating by the physicians. S1C Table shows the frequency per scale.

**Table 2:** The smallest external caliber of the injected vessels.

		Vascular external caliber (mm)					
		Left (n = 5)		Right (n = 5)		Both sides (n = 10)	
Artery	Branch	Mean ± SD	95% CI	Mean ± SD	95% CI	Mean ± SD	95% CI
Superior Thyroid a.							
	Superior laryngeal a.	0.36 ± 0.2	(0.11, 0.61)	0.4 ± 0.16	(0.2, 0.6)	0.38 ± 0.18	(0.26, 0.51)
	Infrahyoid a.	0.18 ± 0.22	(0*, 0.46)	0.16 ± 0.17	(0*, 0.38)	0.18 ± 0.19	(0.04, 0.31)
	Cricothyroid a.	0.21 ± 0.25	(0*, 0.53)	0.14 ± 0.19	(0*, 0.38)	0.18 ± 0.22	(0.03, 0.34)
	Glandular a.	0.1 ± 0.06	(0.03, 0.18)	0.11 ± 0.04	(0.07, 0.16)	0.11 ± 0.05	(0.08, 0.15)
Ascending pharyngeal a.		0.27 ± 0.06	(0*, 0.59)	0.17 ± 0.17	(0*, 0.39)	0.23 ± 0.22	(0.07, 0.38)
Lingual a.							
	Suprahyoid a.	0.07 ± 0.04	(0.01, 0.12)	0.11 ± 0.05	(0.05, 0.18)	0.09 ± 0.06	(0.06, 0.13)
	Dorsal lingual a.	0.2 ± 0.1	(0.07, 0.33)	0.35 ± 0.29	(0, 0.71)	0.28 ± 0.22	(0.13, 0.44)
	Sublingual a.	0.13 ± 0.08	(0.03, 0.22)	0.09 ± 0.05	(0.04, 0.15)	0.11 ± 0.07	(0.07, 0.16)
Facial a.							
	Ascending palatine a.	0.04 ± 0.06	(0*, 0.12)	0.07 ± 0.04	(0.02, 0.12)	0.06 ± 0.06	(0.03, 0.1)
	Tonsillar a.	0.08 ± 0.05	(0.02, 0.14)	0.05 ± 0.05	(0*, 0.12)	0.07 ± 0.05	(0.04, 0.11)
	Submental a.	0.08 ± 0.05	(0.02, 0.14)	0.12 ± 0.12	(0*, 0.27)	0.1 ± 0.09	(0.04, 0.17)
	Inferior labial a.	0.14 ± 0.06	(0.06, 0.22)	0.14	(0.07, 0.2)	0.14 ± 0.06	(0.11, 0.18)
	Superior labial a.	0.17 ± 0.07	(0.07, 0.26)	0.18 ± 0.14	(0, 0.36)	0.18 ± 0.11	(0.1, 0.26)
	Lateral nasal a.	0.14 ± 0.06	(0.06, 0.15)	0.11	(0.09, 0.13)	0.13 ± 0.05	(0.1, 0.16)
	Angular a.	0.11 ± 0.04	(0.06, 0.15)	0.1 ± 0.03	(0.07, 0.13)	0.11 ± 0.04	(0.09, 0.13)
Occipital a.		0.39 ± 0.38	(0*, 0.86)	0.48 ± 0.46	(0*, 1.05)	0.44 ± 0.4	(0.16, 0.73)
Postauricular a.		0.22 ± 0.14	(0.03, 0.33)	0.25 ± 0.28	(0*, 0.6)	0.24 ± 0.21	(0.09, 0.39)
Superficial temporal a.							
	Superficial temporal a.	0.33 ± 0.21	(0.07, 0.59)	0.28 ± 0.03	(0.59, 0.25)	0.31 ± 0.22	(0.15, 0.47)
	Transverse facial a.	0.19 ± 0.13	(0.03, 0.36)	0.26 ± 0.25	(0*, 0.56)	0.23 ± 0.19	(0.09, 0.37)
Maxillary a.							
	Mental a.	0.13 ± 0.07	(0.04, 0.22)	0.1 ± 0.05	(0.04, 0.16)	0.12 ± 0.07	(0.08, 0.17)
	Buccal a.	0.07 ± 0.04	(0.02, 0.13)	0.06 ± 0.04	(0.01, 0.11)	0.07 ± 0.04	(0.04, 0.1)
	Infraorbital a.	0.2 ± 0.04	(0.15, 0.25)	0.15 ± 0.08	(0.05, 0.24)	0.18 ± 0.07	(0.13, 0.22)
Ophthalmic a.							
	Supratrochlear a.	0.12 ± 0.07	(0.03, 0.21)	0.2 ± 0.23	(0*, 0.49)	0.17 ± 0.17	(0.05, 0.29)
	Supraorbital a.	0.13 ± 0.04	(0.08, 0.18)	0.19 ± 0.17	(0*, 0.41)	0.17 ± 0.13	(0.08, 0.26)
	Lacrimal a.	0.04 ± 0.04	(0, 0.09)	0.09 ± 0.05	(0.02, 0.15)	0.07 ± 0.05	(0.04, 0.1)
	Medial palpebral a.	0.11 ± 0.05	(0.04, 0.18)	0.09	(0.03, 0.15)	0.1 ± 0.05	(0.07, 0.14)
	Dorsal nasal a.	0.09 ± 0.01	(0.07, 0.1)	0.1 ± 0.03	(0.05, 0.14)	0.1 ± 0.03	(0.08, 0.11)

*The number is rounded up to zero because the lower limit is in the minus range. However, the vascular caliber can’t be under zero. The minimal data set of this table is in the S1 File, S1B Table.

### Statistical analysis

The primary outcome was the mean of the smallest external calibers of the arteries reached by the injected latex mixture. These were described as mean ± SD and 95% confidence interval. Normality was tested with the Shapiro–Wilk test. Kruskal–Wallis test compared the differences between the left and right artery means. The Likert scale of resemblance to living humans is reported as frequency and percentages of the categorical ratings. Statistical significance was defined as a p-value was < 0.05. Data analysis was performed using PASW Statistics software version 18 (SPSS Inc.).

## Results

Six cadavers were embalmed with the saturated salt solution method. The cadavers were coded from A to F. Table 1 shows their demographic data and latex mixture injection volume. Cadaver F was excluded from the study because of signs of putrefaction at the end of the embalming process.

The dissection started with a skin flap elevation. The consistency of the cadavers was softer than fresh cadavers and living humans. The subcutaneous tissues were preserved, and adipose tissues resembled a living human’s. Muscles were moist and more elastic than those of the conventional embalming method. They were firmer than Thiel’s technique (S1 and S2 Figs). There were no leakages of the injected material from the vessels and no staining of the adjacent tissues. Dissector teams mostly gave ratings that were a high resemblance to living humans for these tissues (Fig 2). The head and neck surgical simulations images are shown in S1A–S1D Fig.

Thyroidectomy and submandibular gland excision were performed. The arterial supply was easily identified. During the neck dissection, the sensations were very close to real-life operations due to the preservation of the adipose tissues (S1 Fig). In contrast, the parotidectomy differed from the real-life experience of surgeons in two ways. First, the parotid tissue was softer than that found in a living human. Second, while the facial nerve trunk was identified, the nerve’s consistency was more delicate than that in a real-life patient (S1D Fig). Fifty-two percent of Likert scale ratings of the resemblance of the head and neck regions to living humans for surgical procedures were four or five rated by five physicians (Fig 2). After finishing the surgical simulations, the eight principal branches of the external carotid artery were followed, and mandibulotomy was performed as the last procedure to find the most distal branches of the lingual artery.

**Fig 3:**
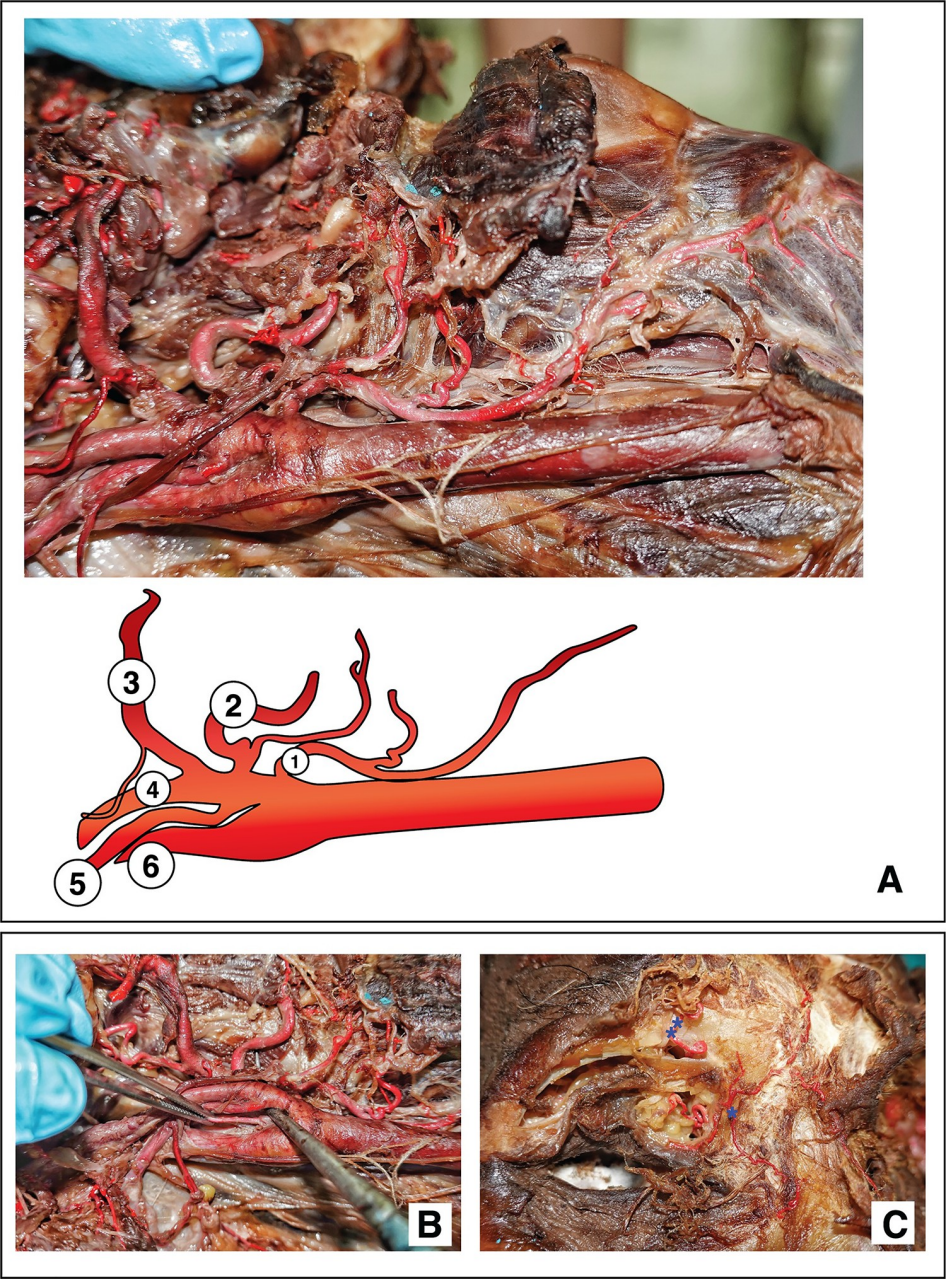
Injection results. (A) External carotid artery branches: (1) superior thyroid artery, (2) lingual artery, (3) facial artery, (4) continuity of the external carotid artery, (5) occipital artery, and (6) internal carotid artery. (B) Ascending pharyngeal artery held by forceps. (C) Dorsal nasal artery or external nasal artery (denoted by one asterisk) and supraorbital artery (denoted by two asterisks).

**Fig 4:**
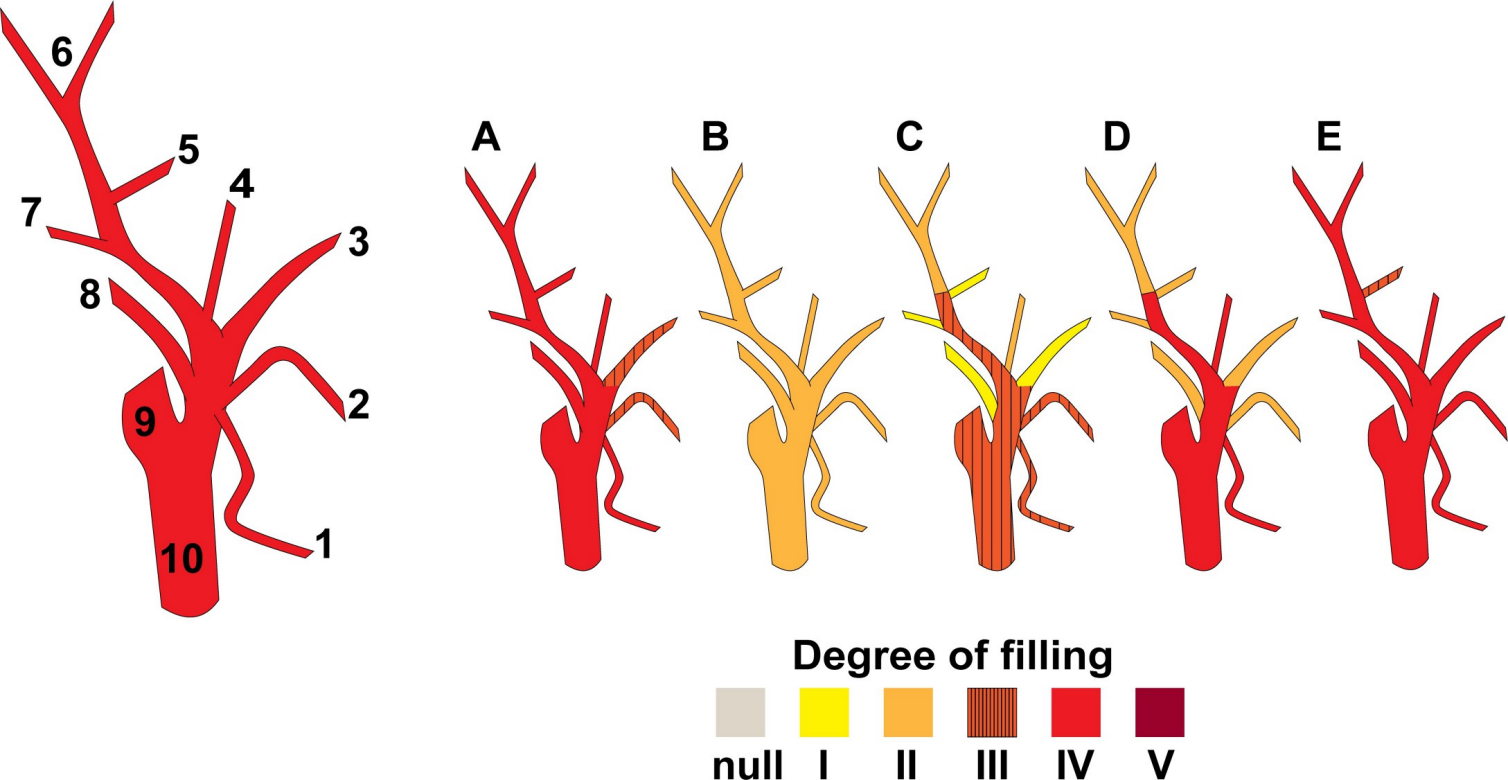
Grading results of the degree of filling of the injected material. (1) superior thyroid artery, (2) lingual artery, (3) facial artery, (4) ascending pharyngeal artery, (5) maxillary artery, (6) superficial temporal artery, (7) posterior auricular artery, (8) occipital artery, (9) internal carotid artery, and (10) external carotid artery. (A) Result of cadaver A. (B) Result of cadaver B. (C) Result of cadaver C. (D) Result of cadaver D. (E) Result of cadaver E. The definitions of the degrees of filling are in the S1A Table. The recorded degree of fill of each artery is in the S1D Table.

Fig 3 illustrates the injection results for the external and internal carotid arteries. The means of the injection completeness visually observed were recorded (Fig 4). The definition of the grading is in the S1A Table. Distal branches with established names were traced [24, 25]. The smallest external caliber of the branch that the latex mixture reached is reported in Table 2, which was the lacrimal artery (mean ± SD, 0.04 ± 0.04 mm). The Kruskal–Wallis test revealed no differences between the mean smallest external calibers in the left- and right-sided arterial branches investigated (p < 0.05).

Craniotomy was performed to investigate the branches of the internal carotid artery. S2A Fig, shows the blood supply of the meninges of cadaver A. We found that the saturated-salt embalming technique partially preserved the brain tissues. The preserved area of cadaver A’s brain is shown in the S2B Fig. In the figure, the specimen shows excellent penetration of the latex mixture throughout the brain tissue.

Each cadaver was wrapped in a polyvinyl chloride sheet and placed on a dissection table with hinged split covers during the dissection and vascular tracing process. The table covers were closed after use. All the tables were in a laboratory and kept at room temperature in Thailand, with an average annual temperature of 28.1°C in 2019 [26]. Over time, the skin and muscles became darker, and there was a decrease in the moisture content of their skin and muscles. Fungus colonies grew on them between four and six weeks after the first dissection.

## Discussion

The steps for the head-neck and vascular labeling were (1) specimen preparation, (2) cannulation, (3) irrigation, (4) color injection, and (5) curing.

### 1. Specimen preparation

In previous studies, the specimens were freshly obtained, defrosted, and embalmed before injection or embalmed after injection [6–8, 12]. In the present study, we used cadavers embalmed with a saturated salt solution. This cadaver type was selected because of its low formaldehyde concentration, low cost, and ability to be stored at room temperature [18, 27]. There have been reports of the successful use of this cadaver type for surgical simulations [18, 28].

Decapitation was previously reported to be a useful method because of its convenience for cannulation, injection, and storage [7]. The suggested cut level was at the fourth or fifth cervical vertebra [7]. However, if the targeted learning area were the neck, such as learning tracheostomy, thyroidectomy, or neck dissection, not having cricoid cartilage, which is usually located at the level of the seventh cervical vertebra, would affect the surgical anatomy [9]. The present study used cadavers that had not been decapitated, which aided leakage control because of the absence of a large raw surface, such as that caused by decapitation. Another advantage was an only minor disturbance to surgical anatomy by the minimally incised targeted area used to access the CCA.

### 2. Cannulation

The CCA is the principal color injection site for the brain as well as the head and neck in decapitated heads. One study performed selective injection via the internal maxillary artery and the CCA, and the CCA method showed better results [29]. Only two studies have published the results of injection techniques in cadavers without decapitation [12, 21].

The reported anatomical landmark for injection in cadavers without decapitation was in the carotid triangle and parallel to the sternocleidomastoid muscle [12]. However, an incision in that area disturbs the anatomical relationships when performing thyroidectomy and neck dissection simulations. In the present study, we cut along the clavicle’s upper border between the two heads of the sternocleidomastoid muscle to enable the important neck anatomical relationships to be preserved (S1 Fig).

Cannulation was the most crucial step for the injection, given that an unsecured injection vehicle might cause damage to a specimen by color leakage [1, 12, 14]. Cutting all sides of the CCA could lead to easy dislodgement of the injection vehicle from the lumen. This study utilized a beveled incision and a 3D-printed adapter to solve this problem. The adapter fitted the CCA lumen perfectly, and its tapered end allowed it to be secured to the blood vessel and the extension tube. There was no displacement of catheters during administrating injections in the present study.

### 3. Irrigation

The purpose of irrigation is to remove clots and debris from the vessel [1]. It also helps to detect vascular breakage points before color injection [7]. Various techniques have been used, such as cold water, room temperature tap water, warm tap water, warm normal saline, as well as a mixture of water and embalming solution. There is no consensus about the irrigation endpoint with either limited volume or irrigating until clear water appears from the contralateral vessel having been suggested [1, 5, 7, 21, 30]. In the present study, we used tap water for irrigation. The irrigated volume was 300 mL, which was twice that of the designated latex mixture injection volume of 150 mL. We hypothesized that too much hypotonic fluid could cause tissue breakage, especially in a cadaver embalmed in a low formaldehyde concentration. Moreover, our preservation method was saturated salt, so that high volume irrigation might have lowered the saturation levels in the preserved tissues.

### 4. Color injection

We chose latex because it was available and is reliable, requiring no preparatory steps except for mixing it with a coloring agent [20, 31]. The unit cost for the locally available latex and ink mixture was 8.75 US dollars in 2021 per kilogram. In contrast, commercial colored silicone injection unit cost is 102.4 US dollars in 2021 per kilogram [32]. A comparative study of vascular injection fluids in fresh frozen and embalmed forearms revealed that the smallest diameter that latex could reach in fresh frozen cadavers was 0.03 mm compared with 0.1 mm in embalmed cadavers [31]. In the present study, we found that the mean smallest external caliber of vessel that could be reached by our latex mixture in saturated salt method-embalmed cadavers was 0.04 mm.

The crucial factor in the color injection step is the injection force. While considerable pressure is needed to push material from bigger to smaller lumens, an excessive force causes vascular breakage. Constant and gentle pressure has been advised [14, 20]. In a forearm injection study, a pressure pump system was utilized, and the researchers used a compressed air pressure system was also used in an oral mucosa vascular study, but the pressure level was not reported [12]. We used a commercial peristaltic pump at its maximum speed to generate a constant injection pressure. Although this method provided good penetration through small-caliber vessels with no vascular breakage, the degree of filling between vessels was not even (Fig 4). The observed inconsistency could have resulted from each cadaver’s vascular properties (such as the degree of atherosclerosis), a clot blockage, or the injection technique. Two studies in which injections were administered through both sides of the CCA had promising results [5, 13]. Thus, two-sided injections with a peristaltic pump force should be further investigated.

### 5. Curing

The final process was the solidification of the latex fluid. The reported waiting time has ranged from 24 to 72 hours [5, 19, 20, 33]. One study injected latex into the heads of fresh cadavers, which were then stored at 5 C°, and they found that the latex was still liquid after s48 hours of storage [5]. The present study found that leaving the latex-injected, saturated salt method-embalmed cadavers at room temperature for 48 hours before dissection was sufficient for the latex mixture to solidify.

### 6. Head and neck surgical simulation

Head and neck surgery is more sophisticated than in the past with a range of new practices, including robotic surgery, endoscopic surgery, and microsurgery. Nevertheless, conventional open surgery is still challenging for learners due to the complex anatomy of this body area. In the present study, we tested the feasibility of a new latex injection technique with saturated salt method-embalmed cadavers. The results were excellent, and the cadaver’s quality was close to a latex-injected, fresh cadaver. The latex-injected cadavers with intact vascular anatomy of the head and neck due to non-decapitation preserved the relevant anatomical relationships for head and neck surgical technique study and simulation.

### 7. Limitations and further study

A strength of the present study is that the method allows an opportunity to study the differences in subcutaneous fat percentage, fat distribution, vascularity, and degree of latex filling between the genders with the preservation technique in the present study since it showed a good quality of the subcutaneous fat preservation.

There were also some limitations. First, while this study used a constant mixing ratio of latex and red ink, the mixture would have been more standardized if its viscosity had been documented. No pressure monitoring system was employed during the injections. Further studies of injection pressures are needed. Second, the critical arteries in the thyroidectomy were the superior and inferior thyroid arteries. Our technique could only label the superior thyroid artery because the mixture was delivered via the CCA only. We were also unable to assess the effect of this injection method on the vertebral artery. This artery has been injected separately from the CCA in decapitated models [6–8, 14]. Further research with whole-body, dyed-latex injection is advisable. Another limitation was the lack of preservation of brain tissues. No prior report indicated that a saturated salt solution inadequately preserved brain parenchyma. In the present study, brain tissues were mostly liquified, which resulted in the inability to trace the anastomosis between the brain’s left- and right-sided arteries. Finally, fungus colonies formed on our cadavers, which is dissimilar to a prior report of no fungal growth following 14 days of embalming using a similar embalming formula [18]. A further study to improve the saturated salt formula is therefore needed. An investigation of the optimum method for storing saturated salt method-embalmed cadavers is also warranted.

## Conclusion

Natural latex mixed with red ink proved to be a low-cost, easy-to-use, reliable material for cadaveric vascular injections. The 3D-printed vascular adapter that we designed along with a peristaltic pump simplified and facilitated the injection process, producing good results. The injection landmark at the clavicle’s upper border is practical for the non-decapitated cadaver because it preserves the crucial anatomical relationships in the neck region for surgical simulations. The saturated-salt embalming method demonstrated promising results, and the latex-injected, saturated salt method-embalmed cadaver quality was close to that of an injected fresh cadaver. The latex-injected, saturated salt method-embalmed cadaver can be used for surgical training models. However, the saturated salt solution embalming method could not prevent the development of fungal infections during a long dissection period of several weeks. Thus, further studies to improve the solution quality are required.

## Supporting information

S1 Fig Head-neck surgical simulations. (A) Finding after skin flap was elevated. Fat in the subcutaneous tissue resembled that of a living human. The sternohyoid muscle was incised to expose the thyroid gland. (B) The thyroid gland was identified; the glandular branch was clearly visible. (C) Neck dissection was performed; the fibrofatty tissue of the neck area was dissectible as in a living human. (D) The facial nerve stem was identified during parotidectomy simulation.
(TIF)

S2 Fig Cerebral vessel injection results. (A) Blood supply of cadaver A’s meninges. (B) Injected cerebral vessels in preserved brain parenchyma.
(TIF)

S1 Table Table of the definition of Fig 4 (S1A Table) and data sets for Table 2 (S1B Table), Fig 2 (S1C Table), and Fig 4 (S1D Table). (DOCX)

S1 File (PDF)
