# Supplementary figures and images for: Latex-injected, non-decapitated, saturated salt method-embalmed cadaver technique development and application as a head and neck surgery training model
Source: PLoS One. 2022 Jan 20;17(1):e0262415. doi: 10.1371/journal.pone.0262415 (PMC8775333; doi:10.1371/journal.pone.0262415)

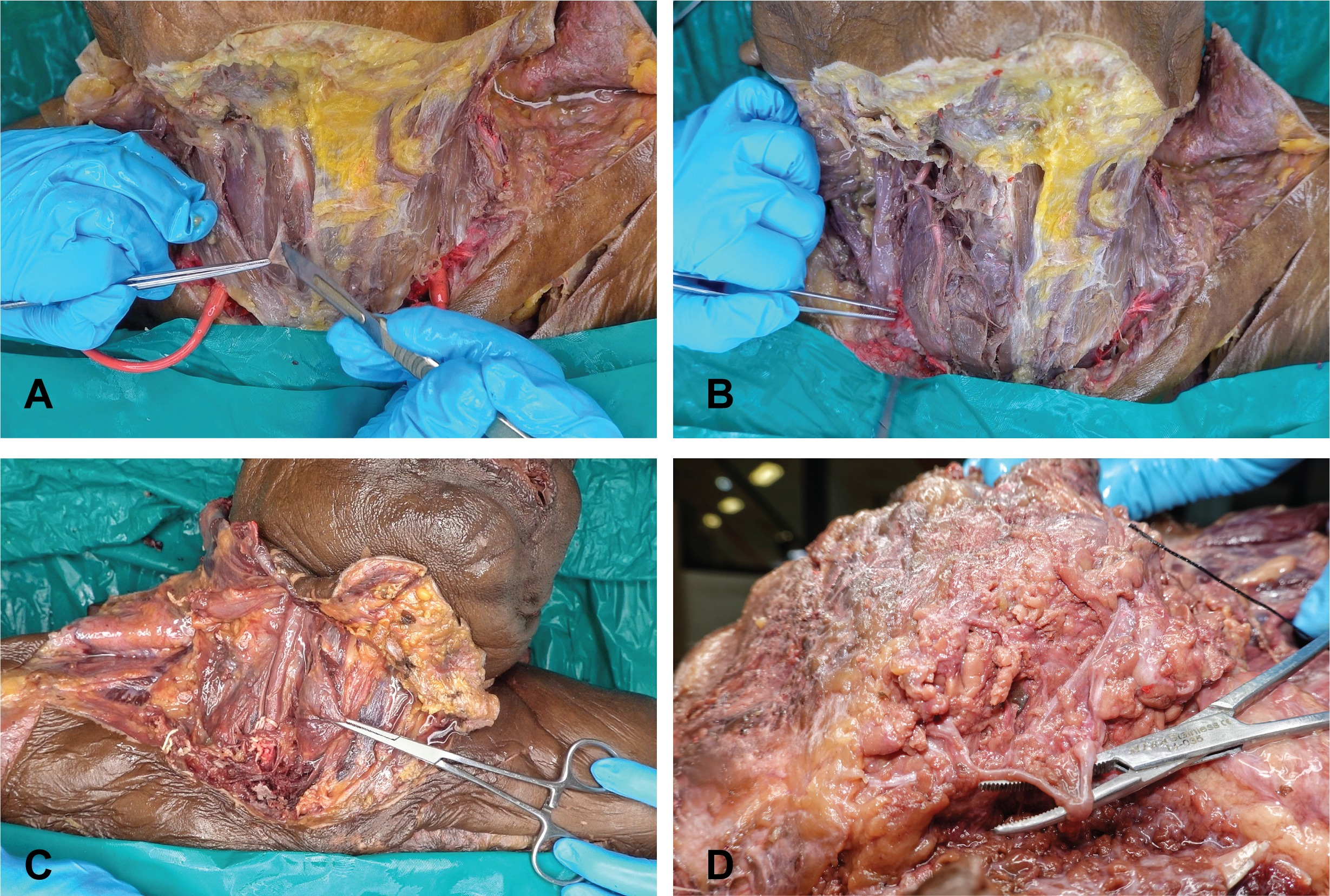

Supplement: S1 Fig — (A) Finding after skin flap was elevated. Fat in the subcutaneous tissue resembled that of a living human. The sternohyoid muscle was incised to expose the thyroid gland. (B) The thyroid gland was identified; the glandular branch was clearly visible. (C) Neck dissection was performed; the fibrofatty tissue of the neck area was dissectible as in a living human. (D) The facial nerve stem was identified during parotidectomy simulation. (TIF) [file pone.0262415.s001.tif]

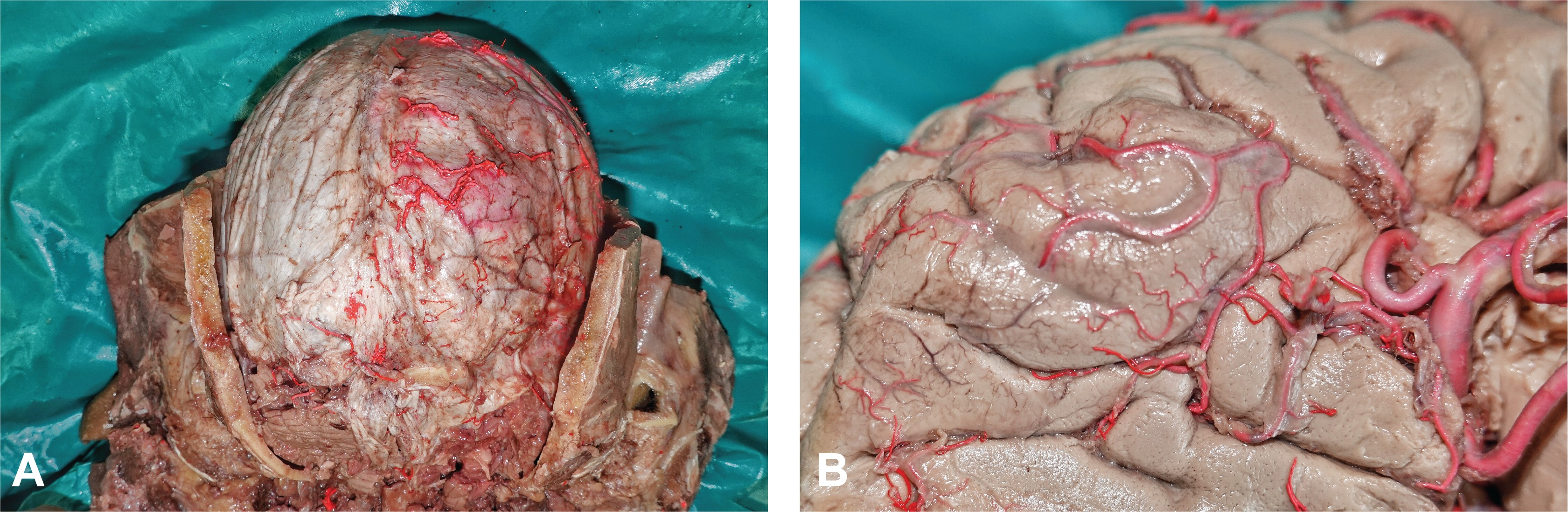

Supplement: S2 Fig — (A) Blood supply of cadaver A’s meninges. (B) Injected cerebral vessels in preserved brain parenchyma. (TIF) [file pone.0262415.s002.tif]
