# Supplementary material for: Latex-injected, non-decapitated, saturated salt method-embalmed cadaver technique development and application as a head and neck surgery training model
Source: PLoS One. 2022 Jan 20;17(1):e0262415. doi: 10.1371/journal.pone.0262415 (PMC8775333; doi:10.1371/journal.pone.0262415)
Supplement: S1 Table — (DOCX) [file pone.0262415.s003.docx]

**PLoS One Supporting Information**

**S1 Table. Table of the definition of figure 4 (S1 Table A) and data sets for table 2 (S1 Table B), figure 2 (S1 Table C), and figure 4 (S1 Table D)**

Article title: Latex injected non-decapitated saturated salt cadavers technique development and application as a head-neck surgery training model.

Authors: Anuch Durongphan1,2*, Songsak Suksantilap1, Nutthanun Panrong1, Aimpat Aungsusiripong1, Apipat Wiriya1, Sasiprapa Pisittrakoonporn1, Witchate Pichaisak3, Benjaporn Pamornpol1*

**Appendix**

**S1 Table A. Definition of Likert-scale of degree-of-fill of the latex injection for figure 4**

**S1 Table B. The recorded smallest external caliber means of the injected vessels of each cadaver. (Data set of table 2.)**

**S1 Table C. Data set of Likert-scale of resemblance to living human-rated by five physicians.**

**S1 Table D. The data were recorded in estimation by consensus of the dissection team. (Data set of Degree-of-fill for figure 4)**

**S1 Table A. Definition of Likert-scale of degree-of-fill of the latex injection for figure 4**

| **Grade** | **Definition** |
| --- | --- |
| Null (0) | No latex in the vascular lumen. |
| I | Latex presents on one side only (left side or right side)  OR  Latex presents on both sides but filling less than or equal to 60% of the vessel’s caliber. |
| II | Latex presents on both sides and fills the vessel to 61-70% of its caliber. |
| III | Latex presents both sides and fills the vessel to 71-80% of its caliber. |
| IV | Latex presents both sides and fills the vessel to 81-90% of its caliber. |
| V | Latex presents both sides and fills the vessel to 91-100% of its caliber. |

**S1 Table B. The recorded smallest external caliber means of the injected vessels of each cadaver. (Data set of table 2.)**

|  | Cadaver | | | | | | | | | |
| --- | --- | --- | --- | --- | --- | --- | --- | --- | --- | --- |
|  | A | | B | | C | | D | | E | |
| Artery | Left | Right | Left | Right | Left | Right | Left | Right | Left | Right |
| Superior laryngeal a. | 0.60 | 0.61 | 0.21 | 0.40 | 0.26 | 0.16 | 0.17 | 0.42 | 0.55 | 0.40 |
| Infrahyoid a. | 0.56 | 0.47 | 0.00 | 0.07 | 0.14 | 0.09 | 0.16 | 0.13 | 0.04 | 0.05 |
| Cricothyroid a. | 0.66 | 0.49 | 0.06 | 0.05 | 0.08 | 0.07 | 0.16 | 0.06 | 0.11 | 0.05 |
| Glandular a. | 0.14 | 0.14 | 0.02 | 0.07 | 0.16 | 0.13 | 0.06 | 0.15 | 0.13 | 0.08 |
| Ascending pharyngeal a. | 0.69 | 0.46 | 0.07 | 0.03 | 0.15 | 0.08 | 0.09 | 0.09 | 0.35 | 0.21 |
| Suprahyoid a. | 0.00 | 0.13 | 0.11 | 0.05 | 0.09 | 0.16 | 0.06 | 0.16 | 0.07 | 0.06 |
| Dorsal lingual a. | 0.33 | 0.18 | 0.18 | 0.43 | 0.27 | 0.28 | 0.15 | 0.81 | 0.07 | 0.07 |
| Sublingual a. | 0.07 | 0.17 | 0.10 | 0.11 | 0.12 | 0.06 | 0.26 | 0.07 | 0.08 | 0.06 |
| Ascending palatine a. | 0.04 | 0.08 | 0.02 | 0.05 | 0.15 | 0.07 | 0.01 | 0.13 | 0.00 | 0.02 |
| Tonsillar a. | 0.07 | 0.01 | 0.11 | 0.09 | 0.08 | 0.12 | 0.13 | 0.04 | 0.01 | 0.01 |
| Submental a. | 0.01 | 0.06 | 0.07 | 0.06 | 0.12 | 0.33 | 0.08 | 0.03 | 0.12 | 0.12 |
| Inferior labial a. | 0.21 | 0.11 | 0.05 | 0.18 | 0.18 | 0.17 | 0.11 | 0.06 | 0.17 | 0.16 |
| Superior labial a. | 0.18 | 0.08 | 0.28 | 0.08 | 0.11 | 0.35 | 0.17 | 0.33 | 0.09 | 0.07 |
| Lateral nasal a. | 0.23 | 0.10 | 0.07 | 0.11 | 0.11 | 0.09 | 0.15 | 0.13 | 0.12 | 0.11 |
| Angular a. | 0.16 | 0.10 | 0.11 | 0.08 | 0.06 | 0.12 | 0.12 | 0.13 | 0.09 | 0.07 |
| Occipital a. | 0.98 | 1.16 | 0.38 | 0.65 | 0.46 | 0.48 | 0.09 | 0.10 | 0.04 | 0.03 |
| Postauricular a. | 0.33 | 0.74 | 0.30 | 0.21 | 0.33 | 0.13 | 0.12 | 0.11 | 0.03 | 0.07 |
| Superficial temporal a. | 0.39 | 0.56 | 0.07 | 0.12 | 0.55 | 0.18 | 0.49 | 0.53 | 0.15 | 0.01 |
| Transverse facial a. | 0.39 | 0.56 | 0.12 | 0.09 | 0.27 | 0.11 | 0.09 | 0.49 | 0.09 | 0.04 |
| Mental a. | 0.12 | 0.06 | 0.11 | 0.07 | 0.26 | 0.15 | 0.11 | 0.07 | 0.07 | 0.15 |
| Buccal a. | 0.00 | 0.00 | 0.10 | 0.05 | 0.10 | 0.10 | 0.08 | 0.05 | 0.09 | 0.09 |
| Infraorbital a. | 0.15 | 0.13 | 0.24 | 0.17 | 0.20 | 0.26 | 0.17 | 0.11 | 0.24 | 0.06 |
| Supratrochlear a. | 0.15 | 0.11 | 0.18 | 0.62 | 0.19 | 0.07 | 0.08 | 0.09 | 0.02 | 0.12 |
| Supraorbital a. | 0.15 | 0.14 | 0.18 | 0.50 | 0.07 | 0.13 | 0.13 | 0.06 | 0.13 | 0.14 |
| Lacrimal a. | 0.00 | 0.15 | 0.07 | 0.13 | 0.03 | 0.05 | 0.09 | 0.08 | 0.03 | 0.02 |
| Medial palpebral a. | 0.12 | 0.11 | 0.15 | 0.16 | 0.09 | 0.07 | 0.03 | 0.05 | 0.17 | 0.05 |
| Dorsal nasal a. | 0.08 | 0.15 | 0.08 | 0.06 | 0.11 | 0.10 | 0.08 | 0.09 | 0.09 | 0.08 |

**S1 Table C. Data set of Likert-scale of resemblance to living human-rated by five physicians.**

|  |  | Physician rate frequency per scale | | | | |
| --- | --- | --- | --- | --- | --- | --- |
| Evaluated item | Cadaver | 1 | 2 | 3 | 4 | 5 |
| Skin | A | 0 | 2 | 1 | 1 | 1 |
|  | B | 0 | 1 | 2 | 1 | 1 |
|  | C | 0 | 0 | 2 | 3 | 0 |
|  | D | 0 | 0 | 0 | 5 | 0 |
|  | E | 0 | 0 | 5 | 0 | 0 |
| Subcutaneous tissue | A | 0 | 0 | 0 | 4 | 1 |
|  | B | 0 | 0 | 1 | 2 | 2 |
|  | C | 0 | 0 | 2 | 3 | 0 |
|  | D | 0 | 0 | 2 | 3 | 0 |
|  | E | 0 | 0 | 2 | 3 | 0 |
| Vessel | A | 0 | 0 | 2 | 1 | 2 |
|  | B | 0 | 0 | 1 | 2 | 2 |
|  | C | 0 | 0 | 0 | 5 | 0 |
|  | D | 0 | 0 | 5 | 0 | 0 |
|  | E | 0 | 0 | 2 | 3 | 0 |
| Nerve | A | 0 | 0 | 3 | 1 | 1 |
|  | B | 0 | 0 | 2 | 1 | 2 |
|  | C | 0 | 0 | 5 | 0 | 0 |
|  | D | 0 | 0 | 2 | 3 | 0 |
|  | E | 0 | 0 | 2 | 3 | 0 |
| Fascia | A | 0 | 0 | 2 | 1 | 2 |
|  | B | 0 | 0 | 1 | 2 | 2 |
|  | C | 0 | 0 | 2 | 2 | 1 |
|  | D | 0 | 0 | 2 | 3 | 0 |
|  | E | 0 | 0 | 3 | 2 | 0 |
| Muscle | A | 0 | 0 | 0 | 4 | 1 |
|  | B | 0 | 1 | 0 | 2 | 2 |
|  | C | 0 | 1 | 1 | 3 | 0 |
|  | D | 0 | 0 | 0 | 5 | 0 |
|  | E | 0 | 0 | 0 | 5 | 0 |
| Head & neck region | A | 0 | 0 | 3 | 1 | 1 |
|  | B | 0 | 1 | 3 | 1 | 0 |
|  | C | 0 | 0 | 2 | 3 | 0 |
|  | D | 0 | 0 | 3 | 2 | 0 |
|  | E | 0 | 0 | 0 | 5 | 0 |
| Pharynx & larynx region | A | 0 | 0 | 3 | 1 | 1 |
|  | B | 0 | 1 | 2 | 1 | 1 |
|  | C | 0 | 0 | 5 | 0 | 0 |
|  | D | 0 | 0 | 2 | 3 | 0 |
|  | E | 0 | 0 | 0 | 5 | 0 |

**S1 Table D. The data were recorded in estimation by consensus of the dissection team. (Data set of Degree-of-fill for figure 4)**

|  | Cadaver | | | | |
| --- | --- | --- | --- | --- | --- |
| Artery | A | B | C | D | E |
| Common carotid a. | 4 | 2 | 3 | 4 | 4 |
| Superior thyroid a. | 4 | 2 | 3 | 4 | 4 |
| Superior laryngeal a. | 4 | 2 | 3 | 4 | 4 |
| Infrahyoid a. | 4 | 2 | 2 | 4 | 4 |
| Cricothyroid a. | 4 | 2 | 2 | 4 | 4 |
| Glandular a. | 4 | 2 | 3 | 4 | 4 |
| Ascending pharyngeal a. | 4 | 2 | 2 | 4 | 4 |
| Lingual a. | 3 | 2 | 3 | 2 | 4 |
| Suprahyoid a. | 3 | 2 | 3 | 2 | 4 |
| Dorsal lingual a. | 3 | 2 | 3 | 2 | 4 |
| Sublingual a. | 3 | 2 | 3 | 2 | 4 |
| Facial a. | 3 | 2 | 1 | 2 | 4 |
| Ascending palatine a. | 3 | 2 | 1 | 2 | 5 |
| Tonsillar a. | 3 | 2 | 1 | 2 | 5 |
| Submental a. | 3 | 2 | 1 | 2 | 5 |
| Inferior labial a. | 3 | 2 | 1 | 2 | 4 |
| Superior labial a. | 3 | 2 | 1 | 2 | 4 |
| Lateral nasal a. | 3 | 2 | 1 | 2 | 4 |
| Angular a. | 3 | 2 | 1 | 2 | 4 |
| Occipital a. | 4 | 2 | 1 | 2 | 4 |
| Postauricular a. | 4 | 2 | 1 | 2 | 4 |
| Superficial temporal a. | 4 | 2 | 2 | 2 | 4 |
| Transverse facial a. | 4 | 2 | 2 | 2 | 4 |
| Maxillary a. | 4 | 2 | 1 | 2 | 3 |
| Mental a. | 4 | 2 | 1 | 2 | 3 |
| Buccal a. | 4 | 2 | 1 | 2 | 3 |
| Infraorbital a. | 4 | 2 | 1 | 2 | 3 |
| Supratrochlear a. | 4 | 3 | 1 | 1 | 3 |
| Supraorbital a. | 4 | 3 | 1 | 1 | 3 |
| Lacrimal a. | 4 | 3 | 1 | 1 | 3 |
| Medial palpebral a. | 4 | 3 | 1 | 1 | 3 |
| Dorsal nasal a. | 4 | 3 | 1 | 1 | 3 |
