# Supplementary material for: Latex-injected, non-decapitated, saturated salt method-embalmed cadaver technique development and application as a head and neck surgery training model
Source: PLoS One. 2022 Jan 20;17(1):e0262415. doi: 10.1371/journal.pone.0262415 (PMC8775333; doi:10.1371/journal.pone.0262415)
Supplement: S1 File — (PDF) [file pone.0262415.s004.pdf]

PLoS One Supporting Information Appendix S1

Article title: Latex injected non-decapitated saturated salt cadavers technique development and application as a head-neck surgery training model.

Authors: Anuch Durongphan1,2*, Songsak Suksantilap1, Nutthanun Panrong1, Aimpat Aungsusiripong1, Apipat Wiriya1, Sasiprapa Pisittrakoonporn1, Witchate Pichaisak3, Benjaporn Pamornpol1*

The following Supporting Information is available for this article:

S1 Table A. Data set of Likert-scale of resemblance to living human-rated by five physicians.

S1 Table B. Definition of Likert-scale of degree-of-fill of the latex injection for Figure 4

S1 Figure 1. Head-neck surgical simulations.

S1 Figure 2. Cerebral vessel injection results.

S1 Table A. Data set of Likert scale of resemblance to living humans rated by five physicians

**Evaluated item (n=10 hemifaces & heminecks)**	**Likert scale frequency (n= 5 physicians)**					
	0	1	2	3	4	5
Skin	0	0	6	20	20	4
Subcutaneous tissue	0	0	0	14	30	6
Vessel	0	0	0	20	22	8
Nerve	0	0	0	28	16	6
Fascia	0	0	0	20	20	10
Muscle	0	0	4	2	38	6
Head & neck region	0	0	2	22	24	2
Pharynx and larynx region	0	0	2	24	20	4

S1 Table B. The smallest external caliber of the injected vessels prior rounded up data.

		Vascular external caliber (mm.)					
		Left (n= 5)		Right (n=5)		Both sides (n=10)	
Artery	Branch	Mean	95% CI	Mean	95% CI	Mean ± SD	95% CI
Superior Thyroid a.							
	Superior laryngeal a.	0.36 ± 0.2	(0.11, 0.61)	0.4 ± 0.16	(0.2, 0.6)	0.38 ± 0.18	(0.26, 0.51)
	Infrahyoid a.	0.18 ± 0.22	(-0.1, 0.46)	0.16 ± 0.17	(-0.05, 0.38)	0.18 ± 0.19	(0.04, 0.31)
	Cricothyroid a.	0.21 ± 0.25	(-0.1, 0.53)	0.14 ± 0.19	(-0.1, 0.38)	0.18 ± 0.22	(0.03, 0.34)
	Glandular a.	0.1 ± 0.06	(0.03, 0.18)	0.11 ± 0.04	(0.07, 0.16)	0.11 ± 0.05	(0.08, 0.15)
Ascending pharyngeal a.		0.27 ± 0.06	(-0.05, 0.59)	0.17 ± 0.17	(-0.04, 0.39)	0.23 ± 0.22	(0.07, 0.38)
Lingual a.							
	Suprahyoid a.	0.07 ± 0.04	(0.01, 0.12)	0.11 ± 0.05	(0.05, 0.18)	0.09 ± 0.06	(0.06, 0.13)
	Dorsal lingual a.	0.2 ± 0.1	(0.07, 0.33)	0.35 ± 0.29	(0, 0.71)	0.28 ± 0.22	(0.13, 0.44)
	Sublingual a.	0.13 ± 0.08	(0.03, 0.22)	0.09 ± 0.05	(0.04, 0.15)	0.11 ± 0.07	(0.07, 0.16)
Facial a.							
	Ascending palatine a.	0.04 ± 0.06	(-0.03, 0.12)	0.07 ± 0.04	(0.02, 0.12)	0.06 ± 0.06	(0.03, 0.1)
	Tonsillar a.	0.08 ± 0.05	(0.02, 0.14)	0.05 ± 0.05	(-0.01, 0.12)	0.07 ± 0.05	(0.04, 0.11)
	Submental a.	0.08 ± 0.05	(0.02, 0.14)	0.12 ± 0.12	(-0.03, 0.27)	0.1 ± 0.09	(0.04, 0.17)
	Inferior labial a.	0.14 ± 0.06	(0.06, 0.22)	0.14	(0.07, 0.2)	0.14 ± 0.06	(0.11, 0.18)
	Superior labial a.	0.17 ± 0.07	(0.07, 0.26)	0.18 ± 0.14	(0, 0.36)	0.18 ± 0.11	(0.1, 0.26)
	Lateral nasal a.	0.14 ± 0.06	(0.06, 0.15)	0.11	(0.09, 0.13)	0.13 ± 0.05	(0.1, 0.16)
	Angular a.	0.11 ± 0.04	(0.06, 0.15)	0.1 ± 0.03	(0.07, 0.13)	0.11 ± 0.04	(0.09, 0.13)
Occipital a.		0.39 ± 0.38	(-0.08, 0.86)	0.48 ± 0.46	(-0.08, 1.05)	0.44 ± 0.4	(0.16, 0.73)
Postauricular a.		0.22 ± 0.14	(0.03, 0.33)	0.25 ± 0.28	(-0.09, 0.6)	0.24 ± 0.21	(0.09, 0.39)
Superficial temporal a.							
	Superficial temporal a.*	0.33 ± 0.21	(0.07, 0.59)	0.28 ± -0.03	(0.59, 0.25)	0.31 ± 0.22	(0.15, 0.47)
	Transverse facial a.	0.19 ± 0.13	(0.03, 0.36)	0.26 ± 0.25	(-0.05, 0.56)	0.23 ± 0.19	(0.09, 0.37)
Maxillary a.							
	Mental a.	0.13 ± 0.07	(0.04, 0.22)	0.1 ± 0.05	(0.04, 0.16)	0.12 ± 0.07	(0.08, 0.17)
	Buccal a.	0.07 ± 0.04	(0.02, 0.13)	0.06 ± 0.04	(0.01, 0.11)	0.07 ± 0.04	(0.04, 0.1)
	Infraorbital a.	0.2 ± 0.04	(0.15, 0.25)	0.15 ± 0.08	(0.05, 0.24)	0.18 ± 0.07	(0.13, 0.22)
Ophthalmic a.							
	Supratrochlear a.	0.12 ± 0.07	(0.03, 0.21)	0.2 ± 0.23	(-0.09, 0.49)	0.17 ± 0.17	(0.05, 0.29)
	Supraorbital a.	0.13 ± 0.04	(0.08, 0.18)	0.19 ± 0.17	(-0.02, 0.41)	0.17 ± 0.13	(0.08, 0.26)
	Lacrimal a.	0.04 ± 0.04	(0, 0.09)	0.09 ± 0.05	(0.02, 0.15)	0.07 ± 0.05	(0.04, 0.1)
	Medial palpebral a.	0.11 ± 0.05	(0.04, 0.18)	0.09	(0.03, 0.15)	0.1 ± 0.05	(0.07, 0.14)
	Dorsal nasal a.	0.09 ± 0.01	(0.07, 0.1)	0.1 ± 0.03	(0.05, 0.14)	0.1 ± 0.03	(0.08, 0.11)

S1 Table C. Definitions of Likert scale categories of the degree of filling of the latex injection for Figure 4

Grade	Definition
Null (0)	No latex in the vascular lumen.
I	Latex presents on one side only (left side or right side)<br>OR<br>Latex presents on both sides but filling less than or equal to 60% of the vessel's caliber.
II	Latex presents on both sides and fills the vessel to 61-70% of its caliber.
III	Latex presents both sides and fills the vessel to 71-80% of its caliber.
IV	Latex presents both sides and fills the vessel to 81-90% of its caliber.
V	Latex presents both sides and fills the vessel to 91-100% of its caliber.

Supplementary figures

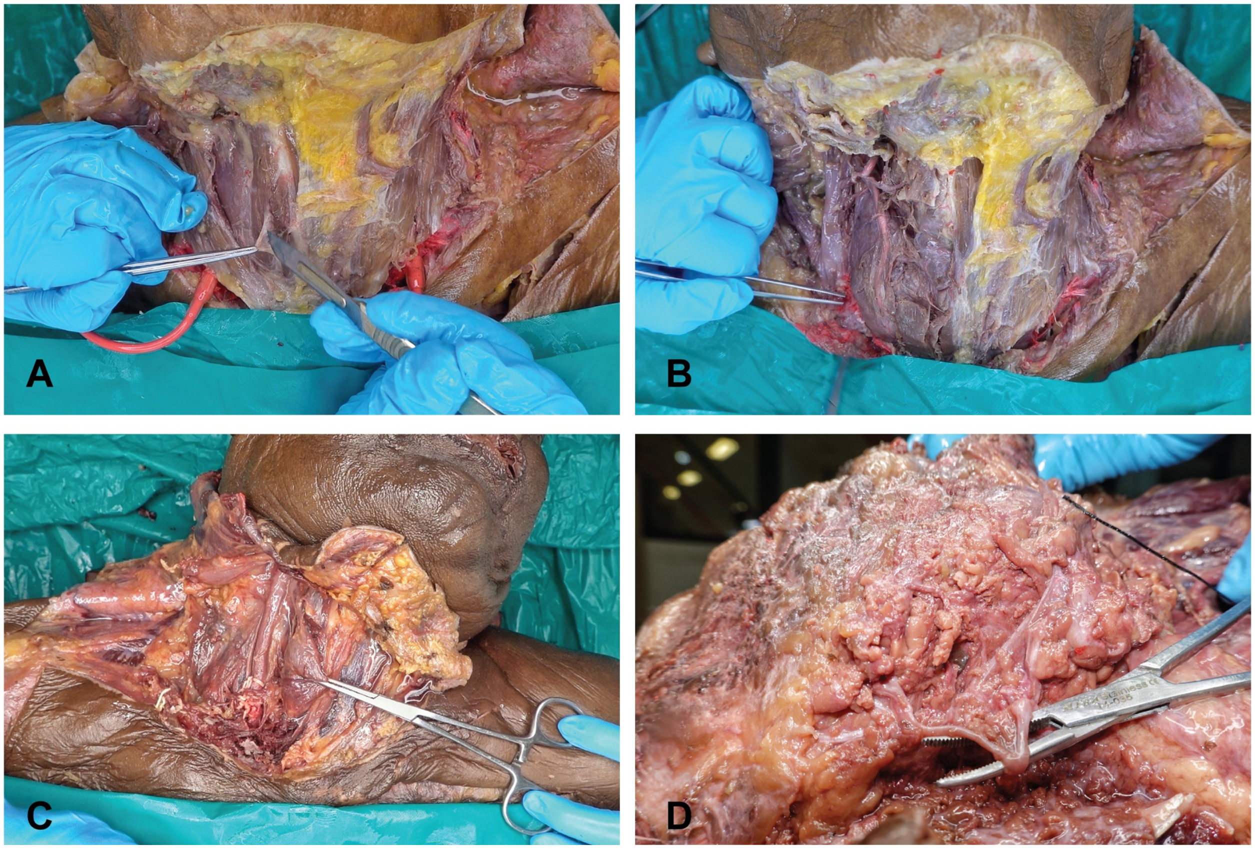

S1 Fig 1. Head and neck surgical simulations (A) Finding after skin flap was elevated. Fat in the subcutaneous tissue resembled that of a living human. The sternohyoid muscle was incised to expose the thyroid gland. (B) The thyroid gland was identified; the glandular branch was clearly visible. (C) Neck dissection was performed; the fibrofatty tissue of the neck area was dissectible as in a living human. (D) The facial nerve stem was identified during parotidectomy simulation.

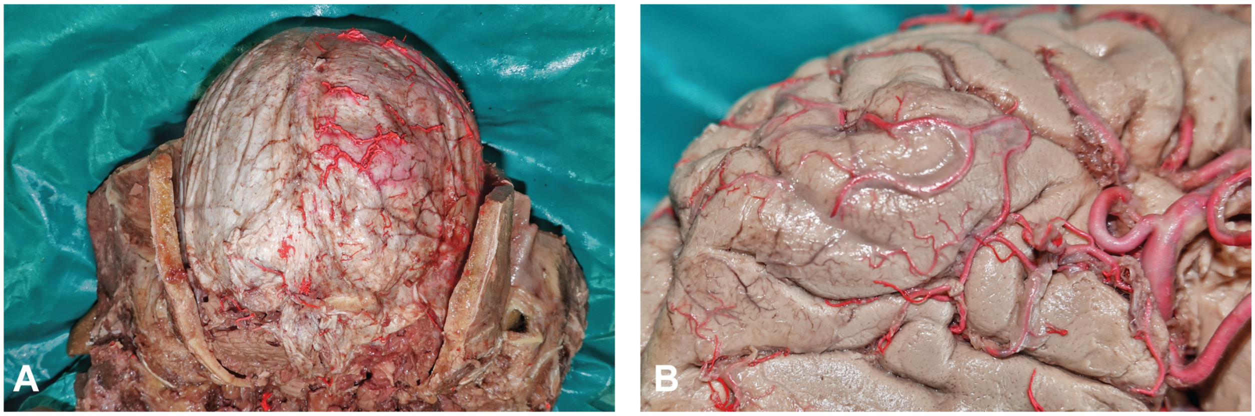

S1 Fig 2. Cerebral vessel injection results. (A) Blood supply of cadaver A's meninges. (B) Injected cerebral vessels in preserved brain parenchyma.
